# Editorial: Women in psychiatry 2022: child and adolescent psychiatry

**DOI:** 10.3389/fpsyt.2023.1248107

**Published:** 2023-07-25

**Authors:** Sara Calderoni

**Affiliations:** ^1^Department of Developmental Neuroscience, IRCCS Stella Maris Foundation, Pisa, Italy; ^2^Department of Clinical and Experimental Medicine, University of Pisa, Pisa, Italy

**Keywords:** psychiatric disorders, neurodevelopmental disorders, children, adolescents, gender bias, women scientists

In the Child and Adolescent Psychiatry field, there is a substantial discrepancy between longstanding recommendations for increase the recruitment of women into child psychiatry research and leadership positions (1), and progress in their active involvement and participation in such activities. Indeed, already in the seventies and eighties of the last century, some studies had brought to light the lower status for women in academia in comparison to male colleagues, including the scientific proficiency in terms of number of publications, research grant awards, citations, and esteem by professional colleagues (2) and their underrepresentation in editorial boards and granting agencies (3). Moreover, a clear gender disparity across faculty rank and among academic leadership positions emerges from a recent U.S. investigation (4). Indeed, 58% of Lecturers, Instructors, and Assistant Professors in child and adolescent psychiatry were women, but this percentage decreased to 53% of Associate Professors and to 31% of Full Professors. In line with the overall disparities among senior faculty, Division Directors were also predominantly men, with only 32% women. Numbers were even most skewed for Named/Endowed Faculty, of whom only 14% were women (4).

Over the past two decades, some changes have occurred to address gender disparities in academic medicine as a whole, such as targeted leadership training programs and networking opportunities for women (5). A virtuous result of when these strategies are applied is represented by the Executive Leadership in Academic Medicine (ELAM) Program Experience at Hahnemann University, which resulted in a significant increase in success rate of women' senior leadership positions (6). Therefore, tailored leadership programs for academics that address gender inequity are of upmost importance and are able to address gender inequity.

In this conceptual framework, the aim of this edition of “*Women in psychiatry 2022: child and adolescent psychiatry*” of Frontiers in Psychiatry is to promote valuable contributions of women in the field. Five articles are included, each featuring women scientists as first or senior author.

Three papers contribute to understanding some parental characteristics that promote improvement of symptom profile and wellbeing in children and adolescents with various neurodevelopmental and psychiatric disorders.

In the study by Flores-Buils and Andrés-Roqueta “*Factors influencing resilience of parents with children with neurodevelopmental disorders: The role of structural language, social cognition, and social support*” the authors investigated the resilience of parents of children with different neurodevelopmental disorder [i.e., autism spectrum disorders (ASD), attention-deficit/hyperactivity disorder, developmental language disorder], and examined which individual factors of the child and which contextual factors of the family support the parental resilience. The authors detected an inverse correlation between parental resilience and the severity of social cognition as well as structural language difficulties of children with ASD. Moreover, formal support from professionals and family association predicted the resilience of the parents of children with any type of NDD.

Iorio et al. in “*Perceived family functioning profile in adolescents at clinical high risk for psychosis: rigidity as a possible preventive target*” investigated the role of the family in a large sample of 111 adolescents at clinical high risk for psychosis (CHR-P), early onset psychosis (EOP), or other psychiatric disorders (no CHR-P). The author found that both mothers and fathers of CHR adolescents did not show a specific profile on perceived familial functioning. However, maternal rigidity was progressively increased from no CHR-P to CHR-P to EOP group, suggesting that this feature could represent a potential target of family intervention, with the aim of promoting the development of a more balanced family style.

Cimino et al. in “*The quality of father-child feeding interactions mediates the effect of maternal depression on children's psychopathological symptoms*” investigated the psychological and interactional functioning of fathers of children with depressed mothers. Specifically, this contribution highlights the negative impact that maternal depression may have on children's emotional and behavioral functioning both at 18 and 36 months. Moreover, the study detected that positive father-child interactions during routine activities that include affective exchanges, could have a protective effect on children's mental health. These findings are of crucial importance for creating both prevention and intervention programs that promote the wellbeing of children in families with depressed mothers.

One article is focused on Mitochondrial disorders (MD), the most frequent metabolic diseases related to genetic mutations in mitochondrial DNA and nuclear DNA. In the study of Riquin et al., “*Neuropsychological features of children and adolescents with mitochondrial disorders: a descriptive case series*” the authors set out to shed light on the levels of intelligence and executive functioning (EF) in a sample of 12 children and adolescents with MD. Results from this monocentric investigation emphasize that MD are heterogeneous diseases whose clinical presentation varies significantly in terms of cognitive capabilities, with the score ranged from 52 to 120 and a particular weakness in the processing speed index. As far as EF, significant difficulties in daily life were signaled by both parents and teachers, suggesting the importance for regular neuropsychological assessments in individuals with MD.

Finally, Budde et al. in “*Influence of identity development on weight gain in adolescent anorexia nervosa*” assessed the impact of identity functioning, measured through the self-report questionnaire “Assessment of identity development in adolescence (AIDA)” on weight gain during inpatient treatment in adolescents with Anorexia Nervosa (AN). Data analyses showed that disturbance of identity development measured by the AIDA questionnaire negatively influences weight gain independently of the weight at admission. For its role in AN illness severity, identity development during treatment should be carefully evaluated and considered during treatment.

This Research Topic supports the advancement of women in the Child and Adolescent Psychiatry field, and is part of continued efforts to achieve diversity, equity, and inclusion in Child and Adolescent Psychiatry (7).

## Author contributions

The author confirms being the sole contributor of this work and has approved it for publication.
